# Incidence, root causes, and outcomes of surgical site infections in a tertiary care hospital in Rwanda: a prospective observational cohort study

**DOI:** 10.1186/s13037-019-0190-8

**Published:** 2019-02-18

**Authors:** Marie Josée Mukagendaneza, Emmanuel Munyaneza, Esperance Muhawenayo, Dancilla Nyirasebura, Egide Abahuje, John Nyirigira, Jean De Dieu Harelimana, Thierry Zawadi Muvunyi, Florence Masaisa, Jean Claude Byiringiro, Théobald Hategekimana, Claude Mambo Muvunyi

**Affiliations:** 1 Infection Control Unit, Kigali University Teaching Hospital, Kigali, Rwanda; 2 Department of Surgery, Kigali University Teaching Hospital, Kigali, Rwanda; 3 Department of Pharmacy, School of Medicine and Pharmacy, College of Medicine and Health Sciences, University of Rwanda, Kigali, Rwanda; 4 Department of Biomedical Laboratory Science, School of Health Science, College of Medicine and Health Sciences, University of Rwanda, Kigali, Rwanda; 5 Department of Clinical Biology, School of Medicine and Pharmacy, College of Medicine and Health Sciences, University of Rwanda, Kigali, Rwanda; 6 Department of Internal Medicine, School of Medicine and Pharmacy, College of Medicine and health Sciences. University of Rwanda, Kigali, Rwanda

**Keywords:** Surgical site infection, Incidence, Microbial etiology, Risk factors

## Abstract

**Background:**

Surgical Site Infections (SSI) are the most reported health acquired infection and common surgical complication in both developed and developing countries. In developing countries such as Rwanda, there is a paucity of published reports on the pattern of SSI, therefore this study aimed at assessing the incidence, risk factors and the antibiotic profile of pathogens responsible of SSI.

**Methods:**

This prospective study included 294 patients admitted between October 10, 2017 and February 12, 2018 to the surgical department of the University Teaching Hospital of Kigali. Patients data were collected using a structured and pretested questionnaire in English version. Regular follow-up was maintained until at least 30 days postoperatively. Samples were collected from suspected wounds and identified using different bacteria culture media. Data were analyzed using Statistical Package for the Social Sciences (SPSS) software word version 20.0. *P*-value < 0.05 was considered statistically significant.

**Results:**

The overall incidence of SSI was 10.9%. The associated risk factors were found to be an increased age, ASA class, wound classification, skills and experience of the surgeon, longer duration of surgery (> 2 h), prolonged duration of hospital stay, blood transfusion and emergency surgery. The most common pathogens isolated were *Klebsiella ssp* (55%), followed by *Escherichia coli* (15%) and *Proteus ssp* (12%), *Acinectobacter* (9%), *Staphylococcus aureus* (6%) and *coagulase-negative staphylococc*i (3%).The pathogens revealed different levels of antibiotic resistance; amoxy-clavilinic acid (98.8%), gentamicin (92.6%), ciprofloxacin (78.1%) and ceftriaxone (53.3%). On the other hand, Amikacin and imipinem were the only two most effective antibiotics for all isolated pathogens with 100% sensitivity.

**Conclusion:**

SSI incidence rate was revealed to be within acceptable international ranges. However, multi drug resistance was seen in half of the isolates leaving clinicians with few choices of drugs for the treatment of patients with SSI. Periodic surveillance of bacteria and antibiotic susceptibility coupled with the implementation of strict protocol for antibiotic administration and operative room regulations are important to minimize the burden of SSI with resistant bacteria pathogens.

## Background

Surgical Site infections (SSI) are the infection following an invasive surgical procedure and are the most frequently reported hospital acquired infections (HAI) [1, 2]. SSI is a type of hospital-acquired infection (HAI) that arises following surgery and it is related to the surgical site [3]. Currently, SSI is defined as an infection that happens within 30 days of the operation if no implant is left in place or within 1 year of operation if an implant is left in place [4].

SSI may result in increased morbidity and mortality, prolonged hospital stay, increased hospital readmissions even reoperation and healthcare costs [5, 6]. It has been reported by numerous studies that diverse surgical specialties were associated with elevated costs next to the development of an SSI in United Kingdom [7–9]. In the United States, for example, SSI is found to be a serious complication with an incidence of 2 to 5% in patients undergoing surgery complicating approximately 300,000 to 500,000 surgeries per year and costing the health-care system upward of $1.6 billion [10, 11]. SSI is the most common surgical complications in both developed and developing countries [12]. Fan Y.et al., 2014 reported 4.5% to be an average incidence of SSI in mainland China from 2001 to 2012 and abdominal surgery to be the most common surgical procedure.

The global estimated prevalence of HAI, at any given time, approximates to 1.4 million. Incidence varies widely across countries and surgical procedures; however, it is estimated to occur in at least 2% of surgeries [10]. In low- and middle-income countries (LMIC), SSI incidence may be approximately up to 4 times higher than in high-income countries [11]. In sub-Saharan Africa, various study results showed that SSI rate is higher than in developed counties with incidences ranging from 11 to 18% [10, 12].

In Rwanda, findings from retrospective study on HAI conducted at University Teaching Hospital of Kigali indicated that SSI account for 76.8% of all HAIs in surgical department and that 35% of children with SSI experienced complications [13]. However, data are lacking the incidences of SSI until 30 days-post operation including discharged patients before that period making difficult to estimate the really burden of SSI. Moreover, the lack of published reports on SSI risk factors, microbial pathogens and their antibiotic profile has negatively impacted the prevention and management of this infection. Therefore, there is a need to explore the extent to, and for which reasons patients develop infection after surgery and the common pathogens involved in SSI in Rwanda.

The aim of the study was to establish the burden of SSI, its risk factors, the etiological bacterial agents associated with SSI and their antimicrobial susceptibility pattern as well as the outcome in patients after surgery in orthopedic and general surgery at University Teaching Hospital of Kigali known as CHUK.

## Materials and methods

### Study design, population and settings

This was a prospective and descriptive study that included 294 patients admitted to the surgical department at Kigali Teaching Hospital between October 2017 and February, 2018. The present study was approved by Kigali Teaching hospital ethics committee and prior to data collection, written informed consent was sought from participants. The target population for the present study was all patients undergoing surgery either as elective or emergency surgical procedures and developed wound infection within the stipulated duration of this study and who were 18 years old and above. Patients who were having another operation within one month proceeding study period were excluded. All patients were evaluated and followed up from the time of admission until the time of discharge and 30 days postoperatively to determine the incidence of SSI.

In this study, all consecutive patients meeting the inclusion criteria were recruited. The structured and pretested questionnaires were used to collect data. Detailed history regarding each case was recorded, such as age, sex, co-morbid conditions, blood transfusion, antibiotic therapy and preoperative hospital stay. The operations were classified as clean, clean contaminated, contaminated and dirty. Other data including associated risks factors (i.e. diabetes, obesity, etc), use of prophylactic antimicrobial agents, the type and duration of surgery. The wound infection was suspected referring to CDC wound infection classification such as superficial infection, deep infection and organ or space of infection.

### Specimen collection and processing

Post-surgical wound swabs or pus aspirates were collected from the clinical infected surgical sites following laboratory standard procedure for specimen collection. Briefly, the surrounding area of the surgical wound was cleaned with 70% ethyl alcohol and excess debris from the wound base removed by irrigating with normal saline before collection of two sterile cotton swabs. Swabs were immediately sent to the microbiology laboratory for analysis, to avoid desiccation and to prevent the growth of some species at room temperature that may obliterate the true pathogens.

After the arrival of the specimen at the microbiology laboratory, swabs or aspirates were inoculated on Mac Conkey and 5% Sheep Blood agar (BA) by rolling the swab over the agar and streaking from the primary inoculums, and aerobically incubated overnight at 37 °C for 24–48 h. The bacteria were identified using standard guidelines. [14]. The antibiotic susceptibility was performed using the standard disc diffusion method following the Clinical and Laboratory Standards Institute (CLSI) guidelines were strictly followed throughout the procedures [15]. After reading the zone diameters, the bacteria were classified as sensitive, intermediate or resistance. Laboratory data (including gram stain, culture results, identification of the bacterial isolates as well as antimicrobial susceptibility) were recorded on a data sheet. Quality control was performed using test strains of *E. coli* ATCC 25 922, *Staphylococcus aureus* ATCC 25923, and *Pseudomonas aeruginosa* ATCC 27853.

### Statistical analysis

All statistical analyses were performed using the Statistical Package for the Social Sciences (SPSS), version 20.0 for Windows (SPSS, Inc. Chicago, IL). First, descriptive statistics, including count and percentage, were used to describe the demographic characteristics of the subjects. The mean and standard deviation were computed for quantitative data variables while Qualitative data were compared using proportion. Bivariate analysis for association between potential risk factors and their potential association with SSI was performed using Chi square (χ2) and Fisher’s exact tests. *P*-value < 0.05 was considered statistically significant.

## Results

The mean age of the patients was 40.41 years (standard deviation [SD], 16.7 years), with a range of 18–90 years. Half of the patients (56.5%) were between the ages of 26–44 years and the rest were almost equally distributed in the age below 25 years of age (15.7%) and above 45 years of age (17.3%). The majority 203 (69%) of the patients were Males and 91 (31%) were females. Half of the patients (55.4%) had completed primary school and 24.8% had finished high school while 16% were illiterates and only 3.7% had attained tertiary education. Participants’ mean BMI 23.4 ± 2.5 kg/m^2^, with the majority of patients (71%) having healthy weight, 26.7% being overweight and only 1.6% obese. Alcohol consumption was found in 37.1% of patients whereas only 10 (3.4%) patients were smokers. Majority of the surgeries (73.8%) were made of orthopedics cases and cases for general surgery and urology were represented 22.4 and 3.7%, respectively.

As shown in Table 3, Majority of the surgeries 211 (71.8%) were performed by licensed Surgeons and 83 (28.25) were performed by Residents in training. There were 142 (48.3%) elective operations and 152 (51.7%) emergency operations. Severity of disease, measured using American Society of Anesthesiologists (ASA) score, ranged from healthy (class I) to severe systemic disease, which is a constant threat to life (class IV). High proportions of patients (71.8%) were found in the class I. Majority of operated patients (98.9%) and all patients who developed SSI were discharge from the hospital while 3 patients died.

### Incidence rate of surgical site infections

The overall incidence of SSI after surgery was 10.2% (34/294) and all of the infections were superficial according to the CDC definition. No infection was identified 30 days after discharged from hospital. Table 1 shows that the incidence of SSI was 10.9% in patient age group below 25 years and 16.9% the age group between 26 and 45 years while no SSI was reported in patient age group above 45 years. The incidence of surgical site infections was 13.3% among male patients compared to 7.7% among females (Table 1). However, the differences in the incidence of SSI among male and female patients were not statistically significant (*p* = 0.165). The occurrence of SSI was similar in orthopedic surgery (12%) and general surgery (12.1%) while there were no cases of SSI in urology surgery. The SSI rate for emergency procedures (19.7%) was higher than the SSI rate for elective procedures (2.8%), a difference, which was statistically significant (p = < 0.001).

**Table 1:** Socio-demographic characteristics of participants who had surgery

	Total (%) (*n* = 294)	N (%) With SSI (*n* = 34)	N (%) Without SSI (*n* = 260)	*p*-value
Age, Yrs, mean = 40.41 ± (16.7)				
≤ 25	46 (15.7)	5 (10.9)	41 (89.1)	0.004
26–45	166 (56.5)	28 (16.9)	138 (83.1)	
> 45	82 (17.3)	1 (1.2)	81 (98.8)	
Gender				
Male	203 (69)	27 (13.3)	176 (86.7)	0.165
Female	91 (31)	7 (7.7)	84 (92,3)	
Level of Education				
None	47 (16)	0 (0)	47 (100)	0.015
Primary	163 (55.4)	19 (11.7)	144 (88.3)	
Secondary	73 (24.8)	12 (16.4)	61 (83.6)	
Tertiary	11 (3.7)	3 (27.3)	8 (72.7)	
Smoking				
Yes	10 (3.4)	2 (20)	8 (80)	0.396
No	284 (96.4)	32 (11.3)	252 (88.7)	
Alcohol				
Yes	109 (37.1)	15 (13.8)	94 (86.2)	0.366
No	185 (62.9)	19 (10,3)	166 (89.7)	

### Risk factors associated with SSI development

Bivariate analysis of risk factors that predict occurrence of SSI is described in the Tables 2 and 3. Several known risk factors for SSI are statistically significant in this study. ASA class II and above had a significantly increased incidence of 22.9% of SSIs compared with ASA class I (7.1%) (OR = 3.9; 95% CI: 1.9–8.1; *P* < 0.001). When surgical procedures were grouped by wound classification, SSI incidence was 8.5 times high in contaminated and dirty wounds compared to clean surgical wounds (OR = 8.5; 95% CI: 2–35.9; *p* = 0.001). Procedures performed by Licensed Surgeons were half times less likely to be associated with SSI compared to procedures performed by Residents (OR = 2.2; 95% CI: 1.1–4.6; *p* = 0.029). The duration of the surgery also had an affect; operations lasting for more than 2 h were associated with SSI more frequently than shorter operations (OR = 2.2; 95% CI: 1.1–4.6; *p* = 0.026). Other factors that predict occurrence of SSI were transfusion during surgery (OR = 6.8; 95% CI: 2.9–16.3; *p* < 0.001) and long hospital stay of more than 14 days due to post –operative complications (OR = 42.3; 95% CI: 16.4–108.9; *p* < 0.001).

**Table 2:** Clinical and procedure characteristics of participants who had surgery

	Total (%) (*n* = 294)	N (%) With SSI (*n* = 34)	N (%) Without SSI (*n* = 260)	*p*-value
BMI kg/m^2^Mean = 23.4 ± 2.5				
< 25	180 (71)	24 (13.3)	156 (86.7)	0.481
25 to < 30	67 (26.7)	6 (9)	61 (91)	
≥30	4 (1.6)	1 (25)	3 (75)	
Diagnosis				
Orthopedic	217 (73.8)	26 (12)	191 (88)	0.473
General	66 (22.4)	8 (12.1)	58 (87.9)	
Urology	11 (3.7)	0 (0)	11 (100)	
Type of Surgery				
Elective	142 (48.3)	4 (2.8)	138 (97.2)	
Emergency	152 (51.7)	30 (19.7)	122 (80.3)	< 0.001
Outcome				
Discharge	291 (98.9)	34 (11.7)	257 (88.3)	0.036
Death	3 (1.1)	0 (0)	3 (100)

**Table 3:** Descriptive data and bivariate analyses of surgical risk factors associated with SSI

	Total (%) (*n* = 294)	N (%) With SSI (*n* = 34)	N (%) Without SSI (*n* = 260)	OR (95% CI)	*p*-value
ASA score					
ASA I	211 (71.8)	15 (7.1)	196 (92.9)	Reference	
ASA II-IV	83 (28.2)	19 (22.9)	64 (77.1)	3.9 (1.9–8.1)	< 0.001
Wound class					
Clean and clean-contaminated	286 (97.3)	30 (10.5)	256 (89.5)	Reference	
Contaminated and dirty	8 (2.7)	4 (50)	4 (50)	8.5 (2–35.9)	0.001
Grade of surgeon					
Surgeon	211 (71.8)	19 (9)	192 (91)	Reference	
Resident	83 (28.2)	15 (18.1)	68 (88.9)	2.2 (1.1–4.6)	0.029
Time of prophylaxis					
≤ 15 min	41 (13.9)	4 (9.8)	37 (90.2)	NA	0.653
15–30 min	248 (84.4)	30 (12.1)	218 (87.9)		
30 min-1 h	5 (1.7)	0 (0)	5 (100)		
Duration of surgery (hours)					
< 2	181 (61.6)	15 (8.3)	166 (91.7)	Reference	
≥ 2	113 (38.4)	19 (16.8)	94 (83.2)	2.2 (1.1–4.6)	0.026
Transfusion					
No	266 (90.5)	23 (8.6)	243 (91.4)		
Yes	28 (9.5)	11 (39.3)	17 (60.7)	6.8 (2.9–16.3)	< 0.001
Number of staff in the room					
≤ 6	150 (51)	18 (12)	132 (88)	NA	
> 6	144 (49)	16 (11.1)	128 (88.9)	NA	0.812
Hospital stay post-operatively (days)					
≤ 14	246 (84)	7 (2.8)	239 (97.2)	Reference	
> 14	47 (16)	26 (55.3)	21 (44.7)	42.3 (16.4–108.9)	< 0.001

Ceftriaxone was the only antibiotic prescribed to all operated patients. The timing of prophylactic antibiotics showed some variations but in the majority of the surgeries (84.4%), antibiotic prophylaxis was administered within 30 min of surgery with an incidence rate of 12.1%; 13.8% within 15 min with an incidence rate of 9.8%; while no cases of infections occurred in 1.7% of surgery with prophylactic antibiotics administered within 30 to 60 min prior to incision. However, the differences in the incidence of SSI in the relation to timing of prophylactic antibiotic were not statistically significant (*p* = 0.653). There was also no significant difference in the incidence of SSI when stratified by the number of people present during the surgery: 12% SSI incidence when 6 people present in the room as compared to 11.1% with more than 6 people (*p* = 0.812).

### Bacterial isolates and their antimicrobial susceptibility profiles

**Fig. 1:**
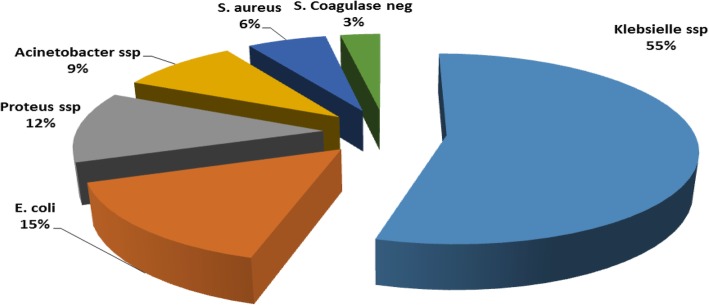
Distribution of pathogens grown on culture of surgical sites

swabs from all patients with SSI were cultured and tested for antibiotic susceptibility. A positive culture was obtained from 32 out of 34 swabs. As it is depicted from Fig. 1, among the cultures with positive growth, the most common organism was *Klebsiella ssp* with an incidence of 55%, followed by *Escherichia coli* (15%) and *Proteus ssp* (12%). Other bacteria isolated were A*cinectobacter* (9%), *Staphylococcus aureus* (6%) and coagulase-negative staphylococci (3%).

**Fig. 2:**
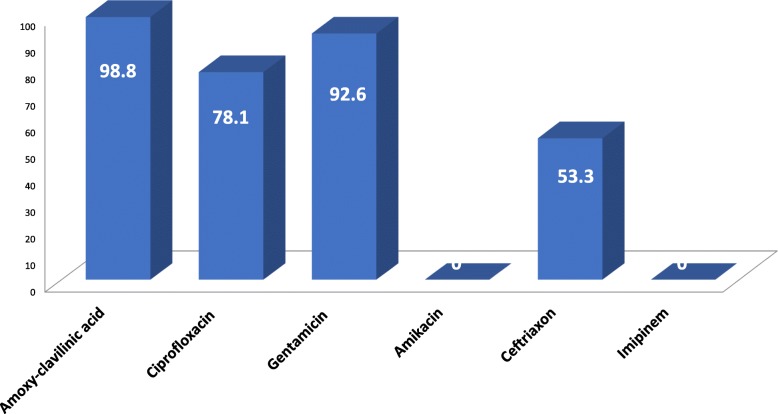
Antibiotic resistance pattern of SSI isolates

As it is demonstrated in Fig. 2, antibiotic sensitivity profiles were reported for the organisms isolated from surgical incision sited in patients with SSI. The pathogens showed very high resistance toward amoxy-clavilinic acid (98.8%), gentamicin (92.6%), ciprofloxacin (78.1%) and ceftriaxone (53.3%). Amikacin and imipinem were the only two most effective antibiotics with 100% of isolates tested sensitive to both antibiotics.

## Discussion

This study has attempted to estimate the burden of SSI by determining not only its incidence and risk factors, but also the etiological bacterial agents associated with SSI and their antimicrobial susceptibility pattern at a referral and tertiary healthcare institution in Rwanda. To our knowledge, this is the first SSI surveillance study in Rwanda, which describes incidence and associated risk factors of SSI using CDC definitions and 30-day follow-up surveillance.

The finding of this study are based on operated cases, of which, the majority (73.8%) were made of orthopedics cases and cases for general surgery and urology were represented 22.4 and 3.7%, respectively. In this study, the overall incidence of SSI was 10.9%, which is comparable to the average of 11.8% in developing countries [4, 16].

The incidence reported here appears to be also of the same magnitude as that reported in a study in India (11%) [17]. However, the incidence rate was higher than those of several developed countries. For example, incidence rates were 1.9% in the United States, 2.2% in Europe, 1.6% in Germany, 1.4% in England, 1.6% in France, and 2.0% in Portugal [18].

The difference observed in the incidence rate of SSI in developed countries compared to developing countries, including Rwanda, may be due to several reasons. These reasons include poor set-up of hospitals (lack of equipment and materials necessary to maintain strict guidelines for asepsis), poor hygiene of patients increasing colonization of skin by bacterial flora, late presentation of patients to healthcare system leading to contaminated wounds, and overwhelmed emergency services due to population burden.

The present findings seem to be consistent with other research which highlighted some SSI risk factors, which are discussed below, such as age, ASA class, wound classification, skill and experience of the surgeon, duration of surgery, blood transfusion and emergency surgery [4, 17]. SSI incidence rate increased with age, which may be due to a poorer immune response and coexistence of other comorbidities. ASA score of 2 or was significantly associated with higher rate of SSI, which may be due to the severity of systemic illness which hinders immunological response in these patients.

SSI incidence rate was higher in contaminated and dirty wounds, not surprisingly, because numerous bacteria, which are the source of the infection, thrive in contaminated/dirty wounds. Consistent with the finding of this study, the skill and grade of the surgeon has been shown to directly affect the incidence of SSI. The more senior and experienced the surgeons, the less likely it was for the patient to develop an SSI.

Two other major risk factors found to be associated with a high incidence of SSI were duration of surgery, which is due to a prolonged exposure of tissue to the environment, prolonged hypothermia and declining levels of antibiotics; long hospital stay, which can be explained by a prolonged stay providing further opportunity for bacterial colonization. Higher rates of SSI in patients who were transfused during surgery can be explained by a reduced Hemoglobin in these patients which may cause hypoxia and impairment in surgical and traumatic tissue oxygenation and healing to favor wound infection.

Emergency surgeries have also been associated with increase in the incidence rate of SSI due like inadequate preoperative preparation, lack of proper control of other medical comorbidities (such as uncontrolled diabetes). Higher frequency of contaminated or dirty wounds in emergency surgeries could also be a contributing factor. Although there was no significant difference in the incidence of SSI in the relation to other potential factors such as timing of prophylactic antibiotic, smoking, BMI, sex, number of staff during, SSI rates seem to be somehow affected by these factors.

In other study, for example, patient factors (smoking, BMI, life style, nutritional status) are associated with resistance of body to germs after operation; similarly, surgical complexity would influence operation duration and exposure possibility [19, 20].

Finally, the microorganisms causing SSI and their antimicrobial resistance patterns were evaluated. *Klebsiella ssp* with an incidence of 55%, followed by *Escherichia coli* (15%) and *Proteus ssp* (12%) were the predominant isolate which is in contrast with the finding of other studies. In these studies, *S. aureus*, has been found to be the predominant cause of SSI, which is can be explained by its presence in the skin as normal flora and can thus enter to deep site during surgery. However, the results of this study agree with the findings of other studies, in which, *Klebsiella ssp* was found to be the predominant bacteria isolated [21, 22].

This difference in the distribution of SSI bacteria may be due to variation in common nosocomial pathogens inhabitant, difference in policy of infection control and prevention between countries and hospitals. Although there are no clear explanations of about high prevalence of Entero-bacterial isolates in the current study, but faecal contamination due to poor personnel hygiene or due to post procedural contamination and outbreaks could be the possible reasons.

High resistance rates to commonly used antibiotic, ranging from 53.3 to 98.8%, were observed in bacterial isolates causing SSI in our study. Not surprising, 53.3% of SSI showed resistance to ceftriaxone, which was prescribed as prophylaxis to all who undergoes surgery. Amikacine and Imipinem were the only effective drugs as 100% of SSI bacterial exhibited very high sensitivity.

The presence of multidrug resistant bacteria isolated in SSI has also been described in other studies in developing countries. This remarkably higher resistance may be due to their easily availability and inappropriate use of the drugs in our hospitals.

## Conclusion

SSI incidence rate (10.9%) was revealed to be within acceptable international ranges. However, multi drug resistance was seen in half of the isolates leaving clinicians with few choices of drugs for the treatment of patients with SSI. There was statistically significant association of SSI with wound class, longer surgery and hospital stay, surgeon experience and grade and emergency surgeries. Therefore, periodic surveillance of bacteria and antibiotic susceptibility coupled with the implementation of strict protocol for antibiotic administration and operative room regulations are important to minimize the burden of SSI with resistant bacteria pathogens.

## Abbreviations

ASA: American Society of Anesthesiologists
CDC: Center for Disease Control
CLSI: Clinical and Laboratory Standards Institute
HAI: Hospital Acquired Infection
LMIC: Low and Middle Income Countries
SPSS: Statistical Package for the Social Sciences
SSI: Surgical Site Infections
